# C9ORF72 hexanucleotide repeat expansion with Alzheimer's disease‐like clinical phenotype: A case report with results from neuropsychology, CSF, FDG‐PET, and PiB‐PET

**DOI:** 10.1002/ccr3.3417

**Published:** 2020-10-27

**Authors:** Zuhal Filikci, Moa Anna Kristina Gustafsson, Otto Mølby Henriksen, Lisbeth Marner, Peter Høgh

**Affiliations:** ^1^ Department of Neurology Zealand University Hospital Roskilde Denmark; ^2^ Department of Clinical Physiology, Nuclear Medicine and PET Copenhagen University Hospital Rigshospitalet Copenhagen Denmark; ^3^ Department of Clinical Physiology and Nuclear Medicine Copenhagen University Hospital Bispebjerg Copenhagen Denmark; ^4^ Department of Clinical Medicine University of Copenhagen Copenhagen Denmark

**Keywords:** Alzheimer's disease, C9Orf72 gene mutation, frontotemporal dementia, genetic testing

## Abstract

A thorough family history and relevant investigation program are essential to settle accurate diagnosis when clinical presentation is atypical or with a mixed picture.

## Graphical abstract

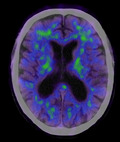

## 1 INTRODUCTION

The C90rf72 genetic mutation is the most frequent cause of familial frontotemporal dementia. This case report highlights the importance of a thorough patient history and relevant investigation program for selection to C90rf72 genetic testing to settle accurate diagnosis, which is essential to providing the best treatment.

Frontotemporal dementia (FTD) is one of the most common early‐onset dementia,^1^ and it has three major clinical subtypes, named the behavioral variant of FTD (bvFTD) and two language variants, semantic dementia (SD), and progressive nonfluent aphasia (PNFA).^1^ Clinically, the picture can be complicated by motor neuron disease (MND), progressive supranuclear palsy (PSP), parkinsonian syndromes, and corticobasal syndrome (CBS).^2^ A pathogenic hexanucleotide repeat expansion of C9Orf72 (chromosome 9: open reading frame 72) has been identified as the most frequent genetic mutation in FTD and MND.^3^

In this paper, we present the case of a 73‐year‐old Danish man with C9Orf72 gene mutation with an Alzheimer's disease‐like clinical phenotype.

## 2 CASE PRESENTATION

A 73‐year‐old man with hypertension was examined for the first time in 2017 and re‐assessed several times over 2‐year period because of a gradual decline in cognitive function and mild behavioral changes since age 60. His spouse reported progressive memory impairment such as forgetting messages and appointments and not being able to remember the location of everyday items in his home, and she complained that he could not recognize familiar faces and places. For example, he got lost in familiar surroundings several times—even within his own home. He had also problems with making a familiar meal due to difficulties in remembering the sequence of actions needed. Furthermore, slowness in movement and poor balance was reported. Changes in social behavior were also observed including loss of social tact and propriety. For example, he started eating before everyone was seated and served. There were no inappropriate comments or changes in food preferences. No visual illusions, hallucinations, dysautonomia symptoms, or insomnia were reported. His symptoms did not fluctuate.

The patient had a negative clinical history for previous psychiatric diagnoses and treatment. There was no history of alcohol or drug abuse. He had worked as an elementary math and music teacher until age 61 and then retired. Interestingly, his mother reportedly at age 64 and grandmother at an even younger age debuted with memory impairment, otherwise no family history of neurological or psychiatric conditions.

## 3 CLINICAL ASSESSMENT

Standard blood analyses were normal. On neurological examination, the patient was alert, partially oriented, and well cooperative. He showed poor insight into his symptoms. There were normal eye movements. In addition to decreased facial expression and a positive pull test, the examination revealed a subtle bilateral and symmetrical wrist rigidity without tremor, fasciculation, bradykinesia, or primitive reflexes. There was neither motor weakness nor any wasting. No MND signs or speech and language impairments were observed.

## 4 NEUROIMAGING

**FIGURE 1:**
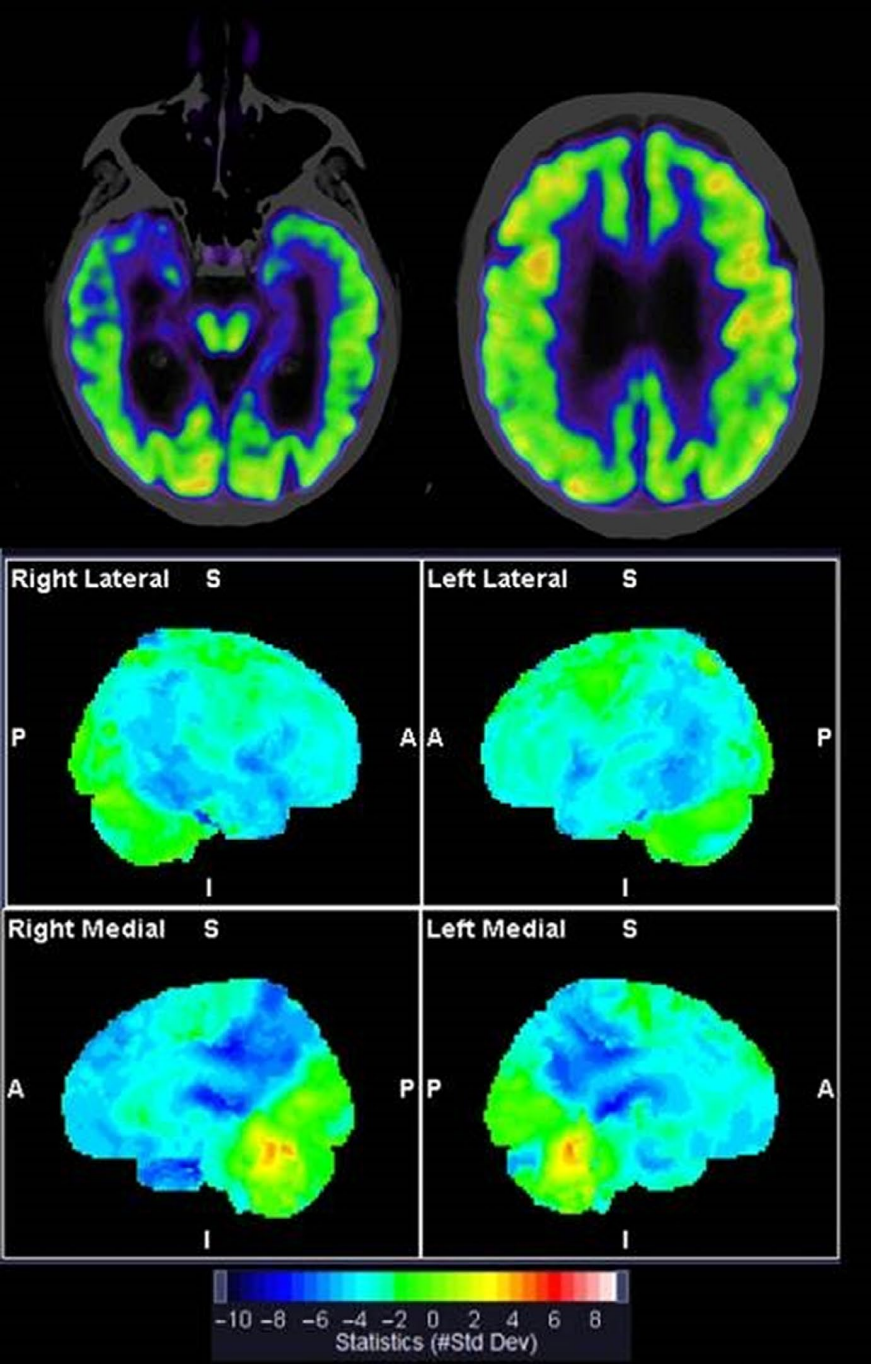
Brain [^18^F]FDG‐PET. Top row shows fused PET and CT axial sections at the level of mesial temporal lobe and posterior cingulate gyrus. Bilateral reductions are present. Below: statistical surface projections normalized to cerebellum and vermis (Syngo.via software, Siemens‐Healthineers). The color bar indicates the standard deviations relative to healthy subjects age 46‐79 y. Characteristic AD‐like pattern with hypometabolism in parietotemporal cortex, posterior cingulate gyrus, and precuneus is demonstrated

The brain computed tomography (CT) scan demonstrated generalized central and cortical atrophy. The 2‐[^18^F]‐fluoro‐2‐deoxy‐D‐glucose positron emission tomography ([^18^F]FDG‐PET) was consistent with a decreased metabolic activity in parietotemporal cortex, in posterior cingulate gyrus and precuneus, mesial temporal lobes, and less pronounced reductions in frontal cortex [Figure 1]. These results were most compatible with AD. Dopamine transporter (DAT) scan, which was performed because of diffuse clinical parkinsonian signs, was unremarkable.

He then underwent cerebrospinal fluid (CSF) analysis which revealed normal values of amyloid β1‐42 (1307, range values >550 ng/L), tau (360, range values <400 ng/L), and phospho‐tau (38, range values <80 ng/L).

**FIGURE 2:**
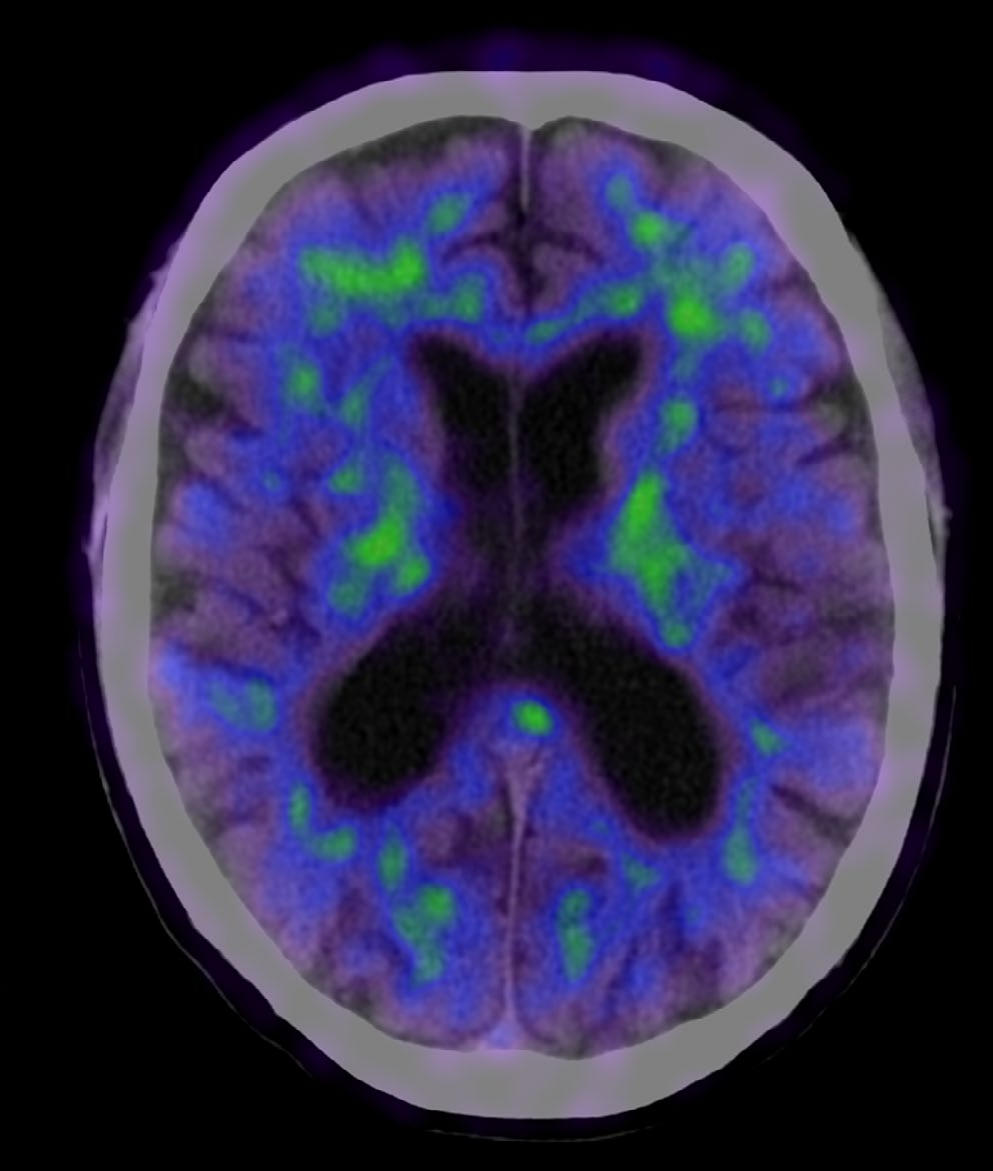
Brain [^11^C]PiB‐PET. The tracer has high affinity for β‐amyloid plaques. The PET scan is overlaid on CT and shows unspecific tracer retention in white matter, but no tracer binding in striate or cortex. Region of interest analysis also showed a normal cerebral to cerebellar cortical standardized uptake value (SUV) ration of 1.02 (normal < 1.5)

As a consequence of discrepancy between clinical profile and normal CSF markers for AD, a Pittsburgh compound B‐PET ([^11^C]PiB‐PET) was conducted. This scan showed no evidence of significant β‐amyloid in the cerebral cortex [Figure 2].

## 5 NEUROPSYCHOLOGY

A brief neuropsychological testing performed in 2017 showed a profile similar to the amnestic type of mild cognitive impairment. A neuropsychological assessment in 2019 showed a significant reduction (more than 1.5 SD) below average for age‐matched controls in all memory tests and several tests of executive functioning. An extremely poor performance on recognition of famous faces was noted. It was concluded that the cognitive profile most resembled that of Alzheimer's disease (AD), but, based on the paraclinical findings (primarily CSF and PiB‐PET), it was suggested that the cognitive profile could also represent an atypical presentation of FTD. Neuropsychological test data from 2019 are shown in Table 1.

**TABLE 1:** Neuropsychological test data from neuropsychological assessment

Cognitive domain/test	Raw scores
Attention/psychomotor speed	
Digit span (forward, backward)	7, 6
Trail making A, B (sec.)	35, 143
Memory	
ADAS‐Cog, immediate recall	0/10^a^
ADAS‐Cog, recognition	4/10^a^
Category cued recall^b^, immediate recall	20/48^a^
Category cued recall, delayed recall	12/48^a^
Category cued recall, recognition	37/48^a^
Rey's complex figure	0/36^a^
Famous faces	14/30^a^
Visuospatial abilities	
Rey's complex figure	31/36
Block design	9/12
Language	
Boston naming test	52/60
Executive functions	
Similarities (WAIS‐IV)	19/36
Stroop color‐word test (sec., errors)	85, 7
Verbal fluency (animals)	9^a^
Verbal fluency (letter: S)	7^a^
Design fluency (figures in 3 min.)	13^a^

^a^ Test performances more than 1.5 SD below expected scores.

^b^ 48 images.

Taken together, these findings led to suspicion of the diagnosis of FTD. Genetic testing was proposed because of a familial history of dementia, and a pathologic expansion was found in C9orf72. The timeline for diagnostic tests is shown in Table 2.

**TABLE 2:** Timeline for diagnostic tests

Time^a^	Diagnostic tests
First clinical examination	Brain CT scan
1 mo later	[^18^F]FDG‐PET Brief neuropsychological testing
5 mo later	Lumbar puncture, Blood analyses DAT scan
17 mo later	[^11^C]PiB‐PET
19 mo later	Neuropsychological assessment
23 mo later	Genetic testing

^a^ Number of months that have passed since the first examination.

## 6 DISCUSSION AND CONCLUSION

In this case, the patient fulfilled clinical diagnostic criteria for AD (executive subtype) according to NIA‐AA/ICD‐10/DSM V criteria.^4^ Imaging with [^18^F]FDG‐PET showed symmetrical hypometabolism in parietotemporal including posterior cingulate and precuneal cortices with less pronounced frontal hypometabolism, in summary most compatible with AD. [^11^‐C]PiB‐PET scan was amyloid‐negative. Thorough neuropsychological re‐evaluation was consistent with AD. In this case, the finding of an amyloid‐negative [^11^‐C]PiB‐PET scan was crucial to the diagnosis.

Familial FTD was subsequently diagnosed by genetic confirmation. As also reported in Ref.,^5^ this patient case confirmed that presenting features of C9orf72 mutation carriers would not always meet diagnostic criteria for probable bvFTD.

Our patient had no psychotic symptoms; however, previous research has reported late‐onset psychotic symptoms of bvFTD secondary to C9orf72 repeat expansions^6^; in this instance, screening for the C9orf72 gene mutation may be suggested, especially when the patient has no previous history of psychiatric disorders.^6^

A family history of dementia and related disorders is reported in 40% of patients with FTD compared with less than 10% in AD.^7^ This case report underlines the importance of a thorough family history, which is an essential component of genetic risk assessment and detailed physical and paraclinical examination when clinical presentation is atypical or with a mixed picture.

## CONFLICT OF INTEREST

The authors declare no potential conflict of interest.

## AUTHOR CONTRIBUTIONS

ZF: contributed to the rationale and patient management, wrote, read, and approved the manuscript. MAKG: provided table with data from neuropsychometry and revised the manuscript. OMH and LM: provide PET readings and prints for figure and revised the manuscript. PH: wrote, read, revised, and approved the final manuscript.

## ETHICAL APPROVAL

The authors have no ethical conflicts to disclose. The authors confirm obtaining written consent from the patient for publication of the manuscript (incl. images, case history and data).
